# A few-shot approach for COVID-19 screening in standard and portable chest X-ray images

**DOI:** 10.1038/s41598-022-25754-6

**Published:** 2022-12-13

**Authors:** Daniel Cores, Nicolás Vila-Blanco, María Pérez-Alarcón, Anxo Martínez-de-Alegría, Manuel Mucientes, María J. Carreira

**Affiliations:** 1 Centro Singular de Investigación en Tecnoloxías Intelixentes (CiTIUS), Universidade de Santiago de Compostela, Santiago de Compostela, Spain; 2 Departamento de Electrónica e Computación, Escola Técnica Superior de Enxeñaría, Universidade de Santiago de Compostela, Santiago de Compostela, Spain; 3 Departamento de Radioloxía, Complexo Hospitalario Universitario de Santiago de Compostela (CHUS), Santiago de Compostela, Spain; 4 Instituto de Investigación Sanitaria de Santiago de Compostela (IDIS), Santiago de Compostela, Spain

**Keywords:** Image processing, Machine learning

## Abstract

Reliable and effective diagnostic systems are of vital importance for COVID-19, specifically for triage and screening procedures. In this work, a fully automatic diagnostic system based on chest X-ray images (CXR) has been proposed. It relies on the few-shot paradigm, which allows to work with small databases. Furthermore, three components have been added to improve the diagnosis performance: (1) a region proposal network which makes the system focus on the lungs; (2) a novel cost function which adds expert knowledge by giving specific penalties to each misdiagnosis; and (3) an ensembling procedure integrating multiple image comparisons to produce more reliable diagnoses. Moreover, the COVID-SC dataset has been introduced, comprising almost 1100 AnteroPosterior CXR images, namely 439 negative and 653 positive according to the RT-PCR test. Expert radiologists divided the negative images into three categories (normal lungs, COVID-related diseases, and other diseases) and the positive images into four severity levels. This entails the most complete COVID-19 dataset in terms of patient diversity. The proposed system has been compared with state-of-the-art methods in the COVIDGR-1.0 public database, achieving the highest accuracy (81.13% ± 2.76%) and the most robust results. An ablation study proved that each system component contributes to improve the overall performance. The procedure has also been validated on the COVID-SC dataset under different scenarios, with accuracies ranging from 70.81 to 87.40%. In conclusion, our proposal provides a good accuracy appropriate for the early detection of COVID-19.

## Introduction

Since the early 2020s, the world has experienced an unprecedented crisis due to the rapid spread of the Coronavirus disease (COVID-19)^1^. Although the number of cases since the beginning of the pandemic has led to notable levels of seroprevalence, it has been proven that herd immunity is not the best way to control COVID-19^2^. Moreover, developed countries have inoculated the population with highly effective vaccines, but there are vast differences among regions^3^, and therefore mobility still plays a crucial role in the spread of the virus^4^. In this regard, all efforts to identify and isolate new cases are key to controlling the pandemic. Therefore, it is highly desirable to speed up the diagnosis of COVID-19 as much as possible.

Currently, the gold standard method for detecting COVID-19 cases is the reverse transcription polymerase chain reaction (RT-PCR) test, which detects SARS-CoV-2 RNA from nasopharyngeal or oropharyngeal swabs with high sensitivity and specificity^5^. The examination of CT or CXR scans has also proved to be a useful method for the screening, diagnosis, and management of patients with COVID-19^6,7^ via identification of abnormalities that are present in patients with the disease. In this regard, different authors studied some of these characteristic visual indicators, which include bilateral and interstitial abnormalities^8^, and ground-glass opacity^9^.

CT refers to a computerized X-Ray imaging procedure that combines scans from different angles, producing 3D radiographic images in which chest findings can be analyzed in great detail. Although the diagnosis of COVID-19 based on CT scans has slightly higher sensitivity than RT-PCR testing^10^, in low-prevalence regions there is a high false positive rate and this test has a low positive predictive value. On the other hand, CXR imaging has some advantages in the context of this global pandemic^11^, such as a quick scanning time, equipment accessibility, and the availability of portable acquisition devices. In practice, both CT and CXR techniques can complement each other when diagnosing COVID-19.

Some studies compared the sensitivity of CXR and RT-PCR to assess the value of this diagnostic method. In^11^, sensitivities of 91% for initial RT-PCR tests versus 69% for CXR were reported. Although later works^12^ reported higher sensitivities—89%, similar to CT analysis—, these results are influenced by the high COVID-19 prevalence during the first peak of the pandemic, and the authors report a disease spectrum skewed to severe cases.

In this context, computer-aided diagnosis methods can help to analyze a large number of scan images in a short time, thus assisting radiologists in effectively diagnosing COVID-19^13,14^. More concretely, Convolutional Neural Networks (CNNs) are particularly interesting due to their capacity to infer the high level features needed to classify the CXR scan images.

In this regard, training robust deep learning (DL) models requires large amounts of data, which caused the research community to put substantial effort in creating datasets to build COVID-19 diagnosis systems^15,16^. However, COVID-19 examples used to build most of these datasets are too heterogeneous and highly biased towards severe cases, and so many recent works reported unusually high sensitivities—far above those achieved by expert radiologists^11^. The clinical value of these models will be limited due to the biased training data, as their performance will drop for detecting patients with low to moderate severity—the true target of these triage systems.

At this moment there are very few high-quality datasets to build robust systems to detect COVID-19 based on CXR scan images^17^. Moreover, the size of the datasets is currently limited, mainly due to the medical staff high workload during all this time. Thus, researchers have had to apply techniques to increase the datasets artificially or specific machine learning algorithms to deal with this data scarcity. In this regard, few-shot learning algorithms have proven to be highly effective^18^.

The image classification problem is traditionally solved by training a deep CNN over a large number of labeled images per each category of interest. Through this learning process, the CNN weights are optimized to detect the most common patterns for each image category. Thus, applying this technique with a limited number of images per category would lead to overfitting as the identified patterns would be biased towards the peculiarities of the few training examples rather than general category traits. To address this issue, few-shot frameworks propose to implement a meta-learning strategy. Instead of identifying the main patterns for each image category, the objective is to learn what makes each category different and, consequently, if two images belong to the same category. In this approach, a set of labeled support images is used to compare with each input query image. The category of the support image with more affinity to the query image is the classification output. As the system learns to differentiate categories in general, the targeting categories in the testing stage can be different from the ones used in the training stage.

In this work, we present a novel approach for detecting the COVID-19 condition in CXR images. It is based on few-shot learning techniques, so it can deal with new small databases. The main contributions of this work are:

- A lung-aware region proposal network that extracts randomly distributed regions of interest targeting the lungs. It removes context information that is not meaningful for the classification task.
- A new cost function specifically designed for this classification problem in which image categories are ordered in different severity levels.
- A novel any-shot experimental setting. In addition to the standard few-shot definition, we propose to use a set of support sets to increase the classification performance when enough data is available. Therefore, our framework can be trained with a limited amount of images per category, but it also benefits from larger datasets.
- A new CXR dataset annotated by expert radiologists that contains COVID positive and negative examples including the severity level for the positive cases. Moreover, negative examples also include patients with COVID-related diseases and other unrelated diseases. To the best of our knowledge, this is the first dataset that contains information about different COVID severity levels and negative examples with other conditions.
- An external validation, with a series of experiments conducted in a publicly available dataset^19^ proving that our method outperforms previous approaches in terms of classification accuracy.

## Related work

Since the pandemic outbreak, the scientific community has been making a great effort to ease and/or improve the COVID-19 diagnosis. However, as in any emerging condition, the available data to develop automated models is still scarce, and the legal issues derived from the massive publication of private medical records hinder the release of high quality public CXR datasets. As it can be seen in Table 1—column Single source—, the approach followed by the researchers was initially the aggregation of datasets of different sources and sizes^15,20^. In further works, curated single-source datasets were published^19,21,22^.

Regarding the different conditions present in the datasets-columns Non-COVID and Other diseases in Table 1, there are some databases focused on COVID-only CXR images^21–23^; other datasets provide also images belonging to healthy patients^19,24^; finally, others included additional images of other conditions, such as pneumonia or lung fibrosis^15,20,25–27^.

In terms of patient positioning when acquiring the CXR image-column View in Table 1, most datasets mix PosteroAnterior (PA) and AnteroPosterior (AP) projections, being each image labeled with the corresponding view. There are, however, some cases where the view is not reported^20,26,27^. The only case where all images were recorded through the same view is COVIDGR, where only the PA view is available. It is worth noting that some datasets report not only the RT-PCR COVID result but also the severity level based on a radiographic diagnosis-column Severity levels in Table 1. For example, BrixIA Covid-19 dataset provides the Brixia score along with the images, and COVIDGR includes a severity label according to the Radiographic Assessment of Lung Edema (RALE) index modified for the COVID-19 quantification^28^.

Overall, the aggregated datasets provide heterogeneous images with a potential bias towards the predominant patient severity and the radiographic view present in each subset. Furthermore, the development of a COVID detection system requires, at least, the presence of both COVID and non-COVID images to establish a reference, so the COVID-only datasets are not useful for this purpose. To the best of our knowledge, COVIDGR is the most complete dataset available at this moment, as it provides high-quality images, a good balance between positive and negative samples, and information of severity levels. However, it does not include images of patients affected by other diseases, which would allow for a more realistic experimentation.

**Table 1:** Main COVID-19 CXR datasets.

Dataset	#Images	Single source	Severity levels	Non-COVID	Other diseases	View
COVID-19 image data collection^15^	761 (305 COVID)	✗	✗	✓	✓	PA/AP/AP supine/ Lateral
Actualmed COVID-19 CXR Dataset Initiative^24^	238 (58 COVID)	✓	✗	✓	✗	PA/AP
Figure 1 COVID-19 CXR Dataset Initiative^25^	55 (35 COVID)	✓	✗	✓	✓	PA/AP/AP supine
COVID-19 Image Repository^23^	243 (243 COVID)	✓	✗	✗	✗	PA/AP
COVIDx^26^	16352 (2358 COVID)	✗	✗	✓	✓	–
BrixIA Covid-19^21^	4703 (4703 COVID)	✓	✓	✗	✗	PA/AP
RICORD COVID-19 dataset^22^	1257 (1257 COVID)	✓	✗	✗	✗	PA/AP
COVID-19 Radiography Database^20^	21165 (3616 COVID)	✗	✗	✓	✓	–
COVIDGR-1.0^19^	852 (426 COVID)	✓	✓	✓	✗	PA
BIMCV-COVID19($+/-$)^27^	10762 (3141 COVID)	✓	✗	✓	✓	–
COVID-SC	1092 (653 COVID)	✓	✓	✓	✓	AP

Regarding the computational models, a variety of approaches for COVID-19 detection in CXR images have been followed, mainly based on deep learning techniques. Some of them relied on popular network architectures, such as VGG^29^, Xception^30^, or CapsNets^31^. Other authors proposed novel architectures aimed specifically at detecting COVID disease. Specifically, Wang *et al.*^26^ proposed COVID-Net, which combines lightweight residual blocks and densely connected layers to enhance the representational capacity while maintaining a high efficient pipeline. In the same way, Ouchicha *et al.*^32^ developed CVDNet, a CNN composed of two interconnected paths with different kernel sizes to capture local and global features.

With the aim of improving the results of the detection models, multiple techniques have been used. Simple image enhancement has proven to increase the COVID-19 detection performance^33^. Ensemble techniques lead to more robust diagnoses through the combination of multiple heterogeneous models^34–36^. To force the detection method to focus only on lung regions, lung segmentation algorithms have been widely used either to preprocess the input images^19^ or to guide the learning algorithm^37^.

Most researchers tried to overcome the problem of working with small datasets. The simplest approach was the use of primitive image transformations to increase the dataset variability^29^. As an alternative, a multi-view representation adding manually designed features to reduce overfitting was explored in^38^. A more elaborated solution in this regard is the inclusion of Generative Adversarial Networks (GAN) to extend the available datasets with synthetically generated images^39,40^. Transfer learning has also been used effectively in most studies by using a network pretrained on a larger dataset, not necessarily composed of similar images^35,40^.

Perhaps the most innovative approach when working with small datasets is the use of few-shot classification techniques^41^, a specific subset of meta-learning algorithms where the learner is aimed at extracting image features which are relevant for differentiating between classes rather than class-specific features. In this way, the methods generalize better to unseen classes. Few-shot has been successfully used for general medical image processing^42^, and also to specifically detect COVID-19 in CXR images^18,43^.

The available methodologies are aimed at detecting a variable number of classes, which mainly depends on the available categories in the input dataset. Although the simplest approach is a binary classification between COVID-19 and non-COVID-19^19,39^, some works proposed a three-class objective—COVID-19, pneumonia and normal^29,35^. Other studies even split the pneumonia class into viral and bacterial to end up with a four-class problem^30,40^.

The performance reported by the different studies has to be cautiously interpreted because of several reasons. For instance, multi-view datasets are commonly biased so that images belonging to severe patients are acquired with an AP CXR for practical reasons. This may lead detection algorithms to focus on device-specific features rather than COVID-19 characteristics^44,45^. Furthermore, most publicly available datasets contain a systematically high number of severe COVID-19 cases, compared to those in mild or moderate condition. As a result, some studies have reported abnormally high COVID-19 detection accuracy and sensitivity, with many works exceeding 95% accuracy. Nevertheless, the generalization of the proposed methods to other datasets is questionable^17^.

## COVID-SC dataset

This work led to the compilation of a high-quality dataset of CXR images. The acquisition, annotation, and storage protocols were defined both by computer scientists of the Research Center on Intelligent Technologies (CiTIUS) and experienced radiologists of the University Hospital of Santiago (CHUS). Both protocols were approved by the Galician Research Ethics Committee (approved on June 23, 2020; approval code: *2020/308 DL-COVIDRX*) and followed the principles of the Helsinki Declaration of 1975 as revised in 1983.

The acquisition process was carried out during 2020 and 2021 by emergency, intensive care and pneumology units. All images were acquired through an AP view by using the same type of portable device, and the RT-PCR was confirmed within 24 hours after the acquisition. Furthermore, the images belonging to positive patients were labeled by six trained thoracic radiologists according to the scoring system proposed in^28^. First, each lung was divided into four regions of equal size. The extension of the consolidations and ground-glass opacity was evaluated according to those regions, resulting in a score between 0 (no regions affected) and 4 (all regions affected). Finally, the score of both lungs was summed up, which yielded a score out of 8. This score was translated into four different levels of severity: P_NORMAL (RALE=0), P_MILD (RALE={1,2}), P_MODERATE (RALE={3,4,5,6}) and P_SEVERE (RALE={7,8}). The negative images were also analyzed visually, establishing three different categories: normal lungs (N_NORMAL), covid-related conditions—like pneumonia or interstitial lung disease—(N_RELATED), and other conditions (N_OTHER).

The total number of collected images was of 1092, distributed in 439 negative and 653 positive. Specific details are given in Table 2.

**Table 2:** Distribution of images in COVID-SC database. F:female; M:male.

RT-PCR result	#	Expert visual diagnosis	#	Label	Age ($\mu \pm \sigma$)	Sex (% F/M)
Negative	439	Normal lungs	229	N_NORMAL	54.0 ± 19.9	54.5/45.5
		Covid-related diseases	77	N_RELATED	70.9 ± 21.8	51.7/48.3
		Other diseases	133	N_OTHER	70.6 ± 18.5	57.6/42.4
Positive	653	Normal lungs	115	P_NORMAL	55.0 ± 20.3	53.3/46.7
		Mild condition	142	P_MILD	66.6 ± 15.9	52.5/47.5
		Moderate condition	294	P_MODERATE	69.4 ± 14.6	69.5/30.5
		Severe condition	102	P_SEVERE	77.8 ± 11.7	55.6/44.4

## Methodology

**Figure 1:**
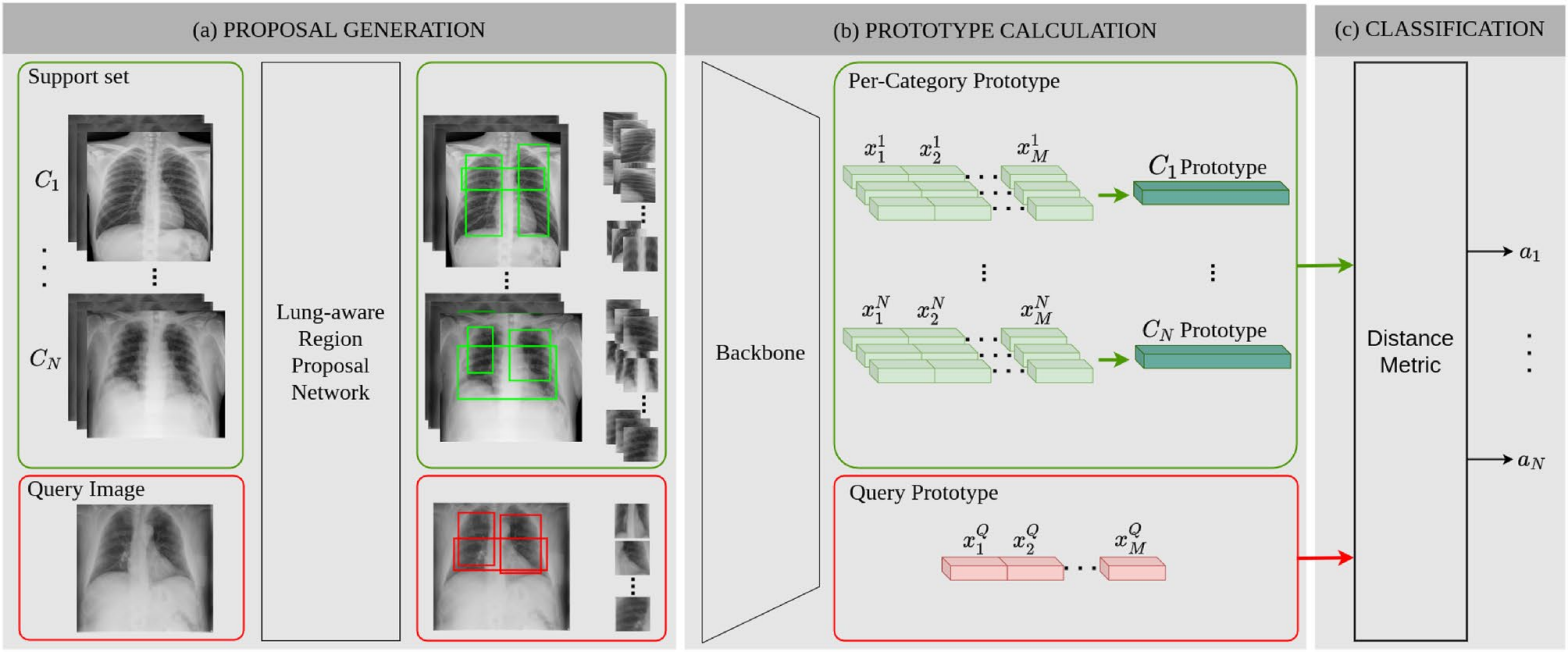
Image classification pipeline for one support set. First, a proposal generator calculates M regions of interest from the images in the support set and the query image, respectively. Then, per-region deep feature maps $x_i$ are calculated for both the support set and query images. These per-region feature maps are concatenated, resulting in a per-image feature map. Support feature maps for every image in each category $C_i$ are aggregated calculating a per category prototype. Finally, the query image feature map (query prototype) is compared with per category support feature maps to obtain the affinity vectors $a_i$.

Figure 1 shows the proposed image classification architecture for automatic COVID-19 diagnosis based on CXR images. Our method can be trained on a limited number of images, resulting in a suitable tool to face the diagnosis of a new disease for which high quality public available data is scarce. Few-shot classification methods are specifically designed to be applied in scenarios in which a traditional image classification approach would fail due to this limited data availability. In a few-shot implementation, each incoming query image is classified according to the similarities with the images of the support set. To this end, per-category prototypes are calculated to compare with the query prototype. These prototypes are based on deep feature maps extracted by a CNN backbone.

First, we propose a lung-aware region proposal network to generate a set of regions of interest targeting the lungs for both the query image and the support set images (Fig. 1a). This allows the network to focus on the most significant regions for COVID-19 diagnosis. More details about this component are given in next subsection.

Then, a CNN backbone is used to extract semantically strong representations for each proposed region in the query and support set images. Per-region feature maps are concatenated to generate a per-image feature map. The network head aggregates features from every support set image that belongs to the same category by calculating the mean feature map (Fig. 1b shows this process for classes $C_1$ and $C_N$). Finally, per-category feature maps are further optimized through gradient descent to calculate strong prototypes that can be used to establish the affinity of each query image to the different classes. This similarity is calculated through the Earth Mover’ Distance (EMD)^46^, which gives the pairwise distance between the prototypes of the query and support regions. Thus, the regions yielding the lower distance are supposed to be the most similar ones.

Traditional few-shot image classifiers use a very small number of support images, with 1-shot and 5-shot settings—one and five images per category, respectively—being the most common scenarios. However, these settings are not realistic in this application as the number of annotated images in every dataset highly exceeds these numbers. Thus, instead of randomly selecting one support set with *k*-shot images per category, we propose to use a group of *S* *k*-shot support sets to reduce the effect of each support image. This process is described in the next *Support set ensemble* subsection.

In this implementation, four ordered object categories are considered for training: negative, mild, moderate, and severe conditions. Therefore, classification errors must contribute differently depending on the distance between the correct and the predicted categories. Taking this into account, we propose a new cost function that includes expert knowledge to guide the training process. Following subsection *Misdiagnosis-sensitive learning* describes this approach.

### Lung-aware region proposal network

The lungs are the most affected organs in the COVID-19 disease, and so they are used as the main radiological diagnostic indicator. Furthermore, patient positioning when acquiring CXR images may be constrained by patient severity or external monitoring devices. Ultimately, this leads to heterogeneous lung position, scale, and rotation within the image, which may hinder the diagnostic capabilities. To overcome this issue, a lung-aware region proposal network is used to focus the classification process in the significant regions of the image regardless of the position of the lungs.

**Figure 2:**
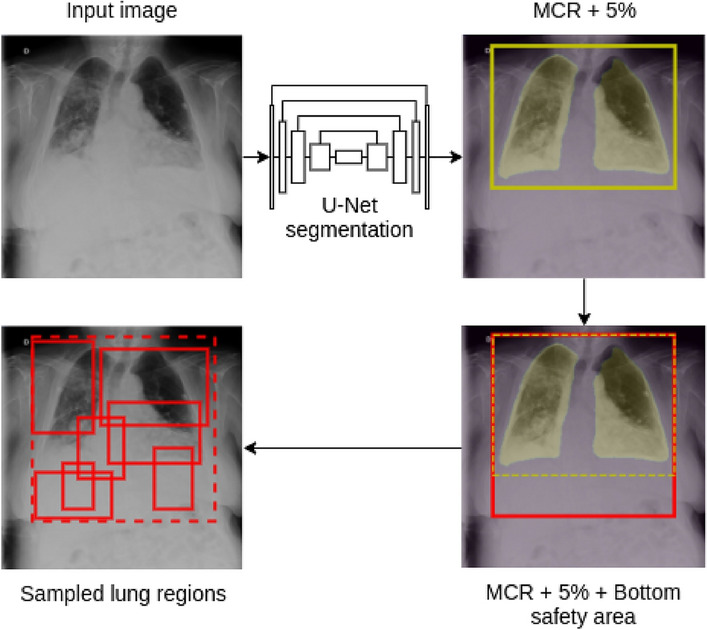
Lung-aware proposal network. First, the lung mask is obtained through a U-net network. The MCR is then calculated and enlarged both by an overall margin of 5% and a bottom margin to get an aspect ratio of 1:1. The final region is used to generate random patches of the original image.

The proposal generation process shown in Fig. 1a represents the first step of the overall architecture. This is shown in detail in Fig. 2. First, a segmentation Deep Neural Network based on the so-called U-net architecture is used to obtain the lung masks^47^. Then, the Minimum Containing Rectangle (MCR) is calculated as the smallest horizontal rectangle that contains all pixels classified as belonging to the lung area. The MCR is then increased by 5% in each dimension for safety reasons. However, the radiopacity of the lung regions which are severely diseased, most often in the lower part of the lungs, may end up disturbing the lung segmentation and so the MCR calculation. To prevent the loss of these areas, the MCR is enlarged on the lower side up to a maximum aspect ratio of 1:1. Then, the refined box is used to constrain a random region generation process, so every image patch lies within these boundaries. The proposal network returns *M* regions of interest for each input image.

### Support set ensemble

Image augmentation is a simple yet effective way of increasing the performance of a machine learning method, especially when few data are available for the training step. It can be applied either at training time or at inference time. While the former increases the variability of the dataset to enrich the learned features, the latter is used to aggregate the predictions of slightly different versions of the same image to boost the overall performance.

Following a similar idea, we select *S* static support sets to conduct the whole test process. Thus, every query image is compared with each support set, resulting in multiple class affinity vectors $a_i^s$, where ${i=}\{1,\ldots ,N\}$ is the class index, and ${s=}\{1,\ldots ,S\}$ is the support set index. In a further step, the probability that a given image belongs to a given class $C_{i}$ is calculated by aggregating the affinity vectors through a weighted voting mechanism:

1 $$\begin{aligned} p_i = \prod _{s=1}^{S} \frac{e^{a_i^s}}{\sum _{j=1}^N e^{a_j^s}} \end{aligned}$$

where $p_i$ is the output probability for the *i*-th class, and $a_i^s$ is the affinity value given by *s*-th support set to the *i*-th class.

### Misdiagnosis-sensitive learning

As in other diseases, the testing systems for COVID-19 show different diagnosis performance. Usually, the cheapest tests are characterized by a high rate of negative patients testing positive, what is also known as a high number of false positives. Although the maximum accuracy is always desired, these results yield a reasonably good triage approach where the positive results can be refined in a further step with high accurate, yet more expensive, testing procedures.

Our objective is to solve a binary classification problem, i.e., to distinguish between negative and positive COVID-19 CXR images. To improve the training process, we leveraged the additional categories available in COVID-SC and COVIDGR-1.0 datasets. Regarding the former, we initially split the negative/positive images into seven classes, according to the expert visual diagnosis explained in Table 2. However, both negative and positive images with no visual affection may be very similar. Furthermore, the images of negative patients with COVID-related diseases, like pneumonia, can be confused with positive images. To overcome these problems that could confuse the network learning, both the negative patients affected by COVID-related diseases and those positive patients with no visual lung affection are not included in the classifier training step—they are only taken into account for testing the classifier. As a result, a 4-class training setup problem is defined with the following classes: N_NORMAL, P_MILD, P_MODERATE and P_SEVERE. As for the COVIDGR-1.0 dataset, we used the four available categories, which exactly match those finally used with COVID-SC.

With a point to improve the actual automatized triage systems, the diagnosis procedure developed in this work aims at minimizing the loss of positive COVID diagnosis through a misclassification penalty. This term depends both on the visual distance between the classes and the clinical cost of each wrong classification. Intuitively, the greatest penalization should be given to a COVID patient with severe lung affection classified as a negative patient, as both the visual distance between classes and the clinical cost of a false negative are maximum. On the contrary, a COVID patient with moderate lung affection classified as a positive patient with severe affection should be penalized to a lesser extent, as both classes are very similar visually and the clinical cost is zero.

To include all this expert knowledge, we introduce a cost matrix *M* designed and validated by experienced radiologists in the original formula (see Table 3) of Cross Entropy Loss. The value $M_{ij}$ represents the extra cost of a patient belonging to the *i*-th class classified within the *j*-th class. As can be seen, the greatest penalties are given to patients with mild and severe COVID conditions who are incorrectly classified as COVID negative, as these are the cases with the highest cost in case of misclassification.

**Table 3:** Cost matrix *M* included in the new loss function (2).

		Predicted			
		Negative	Mild	Moderate	Severe
Real	Negative	0	0.1	0.2	0.3
	Mild	0.3	0	0.1	0.2
	Moderate	0.4	0.1	0	0.1
	Severe	0.5	0.075	0.025	0

Therefore, the cost of classifying a patient of the *i*-th class is:

2 $$\begin{aligned} {\mathscr {L}}_i = -log(p_i) + \sum _{j \in C'} \big [ -log(1-p_j) M_{ij} \big ]\quad , \quad {C'=}C\setminus \{C_i\} \end{aligned}$$

where $C'$ is the set consisting of all classes except the class $C_i$.

### Ethical approval

This study was performed in line with the principles of the Declaration of Helsinki. Approval was granted by the Galician Research Ethics Committee (approval date: June 23, 2020; approval code: *2020/308 DL-COVIDRX*). As it is mandatory in this approval, informed consent was obtained from all subjects.

## Experiments

### Datasets

We analyze the performance of our framework in a wide range of scenarios using the new COVID-SC dataset. The availability of detailed information regarding severity level and other non-COVID and COVID-related findings allows us to evaluate the classification accuracy in different challenging real life use cases.

Moreover, we also evaluate our model in the publicly available COVIDGR-1.0 dataset^19^ to compare the outcome of our system with state-of-the-art automatic COVID-19 diagnosis systems based on CXR images. This dataset contains 852 labeled images with 426 positive examples and 426 negative examples. It also provides information about the severity level following the RALE index. Although this dataset does not have images with other findings, it contains RT-PCR positive examples labeled as normal by experts. For other methods, we provide the results reported in^19^. To perform a fair comparison, the same validation setup—5 times repeated 5-fold cross-validation—was used to evaluate our method.

### Evaluation metrics

In the experiments, we report the binary classification performance using the following metrics to evaluate and compare our model with the state-of-the-art:

3 $$\begin{aligned} specificity= & {} N\_recall = \frac{TN}{TN+FP} \end{aligned}$$

4 $$\begin{aligned} sensitivity= & {} P\_recall = \frac{TP}{TP+FN} \end{aligned}$$

5 $$\begin{aligned} N\_precision= & {} \frac{TN}{TN+FN} \end{aligned}$$

6 $$\begin{aligned} P\_precision= & {} \frac{TP}{TP+FP} \end{aligned}$$

7 $$\begin{aligned} F1= & {} 2\cdot \frac{ precision \cdot recall }{ precision + recall } \end{aligned}$$

8 $$\begin{aligned} accuracy= & {} \frac{TP+TN}{TP+FP+TN+FN} \end{aligned}$$

being TP the number of true positives, TN the true negatives, FP the false positives and FN the false negatives. It is worth noting that F1 metric can be calculated either for positive or negative categories by using positive or negative precision and recall metrics, respectively.

Achieving a high sensitivity (Eq. 4) is crucial for any triage system, as it means that a high percentage of positive cases are detected. However, a trade-off between specificity (Eq. 3) and sensitivity (Eq. 4) is required to minimize the false positives rate. Otherwise, a system that classifies almost every example as positive would be useless. The F1 (Eq. 7) metric also takes into account this balance as it is based on the recall and the precision for each specific category.

The accuracy (Eq. 8) represents the global classification precision measuring the percentage of images correctly classified. Therefore, it is also a useful metric to compare different classification approaches.

### Implementation details

The feature extractor of the few-shot architecture was implemented as a ResNet-12 pretrained on the miniImageNet dataset^41^. Input image regions were generated following the method described in previous subsection *Lung-aware region proposal network* and rescaled to $128 \times 128$ pixels. The number of sampled regions per image (*M*) was set to 9. The support set size was set to 5 images per category (5-shot) by default. One randomly selected support set was used in each meta-learning iteration (episode), while 5 static support sets, with 5 images per category each, were used in validation and test. Support images for validation and test were randomly selected from the training set keeping the same support sets for both tasks. We performed a 5-fold cross-validation for all the experiments, showing both the mean and the standard deviation for every metric.

The U-net model for lung segmentation was trained on both the Montgomery and SCR datasets^48,49^. The segmentation results on these public databases exceeded 95% both in terms of Intersection over Union and Dice score. The performance of the segmentation network was also validated on both COVID-SC and COVIDGR-1.0 datasets through a visual examination by expert radiologists.

With the aim of increasing the diagnosis performance, the image categories which can potentially lead to confusion with respect to other opposite-diagnosis categories were removed from the training set. This affects the classes N_RELATED and N_OTHER—which can be confused with every positive class— and P_NORMAL—which is highly similar to N_NORMAL. To keep a realistic evaluation scenario, however, those classes were not removed from the validation or test sets.

We trained our network following a meta-learning approach in which the network learns to differentiate images from different categories rather than focusing on the specific features of each category. The maximum number of episodes was set to 5000 with a validation step each 50 episodes. The initial learning rate was set to $0.5 \times 10^{-3}$ with a reduction by a factor of 0.5 after every 500 episodes. The best validation iteration was selected measuring the validation performance *VP* as:

9 $$\begin{aligned} VP = \alpha * sensitivity + \beta * accuracy \end{aligned}$$

Therefore, we maximize both the accuracy as a metric of global classification error, and the sensitivity which is crucial for any triage system. Optimal values for $\alpha$ and $\beta$ parameters were calculated through a Grid Search setup on the training set, being 0.3 and 0.7, respectively.

### Ablation studies

We conducted a series of ablation studies to assess the influence of each novel component on the final classification accuracy. These experiments were performed on just one cross-validation repetition from the COVIDGR-1.0 dataset to keep a reasonable experimentation time.

Table 4 shows the classification accuracy and the standard deviation for the different scenarios. The baseline accuracy, without the inclusion of the proposed improvements, is almost 76.5%, being the standard deviation over 7%. First, the addition of support ensembling increases the mean accuracy by around 1.5% while the standard deviation is decreased by more than 2%. Second, the misdiagnosis-sensitive learning has a less noticeable impact, yet it also improves the overall accuracy and reduces its variability. Finally, the lung-aware region proposal network boosts the accuracy by 1.5% and reduces the standard deviation to only 2.82%, making the system outcome more predictable. Overall, the proposed additions yield an accuracy increase of 3% and a deviation reduction of 4.48%.

**Table 4:** Ablation studies.

Support set ensemble	Misdiagnosis-sensitive learning	Lung-aware	Accuracy
✓			77.93 ± 5.03
✓	✓		78.05 ± 4.56
✓	✓	✓	79.48 ± 2.82

### Results

Table 5 shows the results of our system on the COVID-SC dataset. We defined a baseline experiment in which we removed from the test set RT-PCR positive examples labeled as normal by experts (P_NORMAL) and negative examples with other COVID-related conditions (N_RELATED) and non-related conditions (N_OTHER). In this ideal scenario, we achieve a 87.40% accuracy with a 91.42% sensitivity and 77.95% specificity.

**Table 5:** Performance on different COVID-SC testing setups: baseline experiment and, in following rows, the effect on performance of adding other categories.

	Specificity	N_Precision	N_F1	Sensitivity	P_Precision	P_F1	Accuracy
base categories N_NORMAL P_MILD P_MODERATE P_SEVERE	77.95 ± 5.14	79.93 ± 4.78	78.64 ± 1.68	91.42 ± 3.07	90.80 ± 1.73	91.05 ± 0.92	87.40 ± 1.10
+ N_OTHER	63.03 ± 4.72	83.59 ± 3.95	71.75 ± 3.66	91.59 ± 2.33	78.70 ± 2.07	84.64 ± 1.69	80.11 ± 2.26
+ N_RELATED	62.40 ± 7.57	79.32 ± 9.32	69.05 ± 5.02	89.78 ± 6.75	80.95 ± 2.44	84.96 ± 3.11	79.85 ± 3.52
+ P_NORMAL	72.63 ± 11.87	59.66 ± 5.04	64.89 ± 5.68	82.49 ± 4.89	89.89 ± 3.65	85.86 ± 2.29	79.93 ± 2.91
+ N_OTHER + P_NORMAL	62.44 ± 10.44	63.30 ± 2.06	62.25 ± 5.58	79.71 ± 4.58	79.64 ± 3.21	79.49 ± 0.96	73.57 ± 1.30
+ N_OTHER + P_NORMAL + N_RELATED	58.96 ± 8.80	65.81 ± 3.99	61.54 ± 3.48	78.80 ± 6.27	74.40 ± 2.87	76.28 ± 1.69	70.81 ± 0.87

We designed a series of experiments to evaluate how more challenging real world scenarios affect the performance of our system in comparison with the ideal baseline. The same network weights were used for all experiments.

Adding negative cases with other conditions (N_OTHER) mostly affects the specificity. Thus, the precision in the positive class also drops around 10%, proving that more negative images are being classified as positive. In fact, the results are slightly worse in the COVID related case, with around 2% less sensitivity. However, the fact of adding examples with unseen conditions has a bigger impact than confusing negative patients with positives due to the visual similarities between their previous conditions and COVID-19.

On the other hand, adding P_NORMAL hinders the classification in the positive class. In this case, the sensitivity decreases to 82.49% and the precision in the negative class drops to 59.66%. Thus, as these images are more visually similar to negative examples than the positive ones, the network tends to classify them as negative.

Finally, the last row in Table 5 represents the most challenging setting considering the complete test set. The sensitivity drops from 91.42 to 74.40% while the accuracy decreases around 17% getting 70.81%. It proves the necessity of new datasets such as COVID-SC to evaluate automatic triage systems in more realistic scenarios.

Table 6 shows the results by severity level. All severe patients, 95.96% patients with moderate condition, and 78.50% patients with mild condition are classified as positive. These findings prove that our method is not only capable of correctly classifying patients with moderate and severe conditions, but it also achieves good results in the P_MILD subset. In this table, we consider P_NORMAL as a different severity level to independently evaluate the results in this challenging subset. This subgroup contains positive cases labeled as negatives by experts. Therefore, the visual information given by CXR images is not sufficient to correctly detect these positive cases. Moreover, the results obtained in the different COVID-negative categories prove that the inclusion of patients with other radiologically visible diseases (both N_OTHER and N_RELATED categories) can affect the performance of COVID detection negatively. This negative effect is more noticeable in the N_RELATED case, in which the radiological findings are very similar to those of COVID-positive examples.

Table 7 shows the performance comparison between a conventional CNN architecture—ResNet-50— and the proposed approach in a testing setup where all four levels of severity are included. As can be seen, the architecture proposed in this work outperforms ResNet-50 in every single comparison. The most notable differences were obtained in the metrics related to the classification of COVID-positive patients. Specifically, the sensitivity was improved from 71.62 to 91.42% and the precision of the positive class was boosted from 62.81 to 90.80%. In this regard, we consider that our approach is more reliable as an automatic triaging system, as it is capable of detecting more COVID-positive patients.

**Table 6:** Accuracy results by category on COVID-SC.

N_NORMAL	N_OTHER	N_RELATED	P_NORMAL	P_MILD	P_MODERATE	P_SEVERE
82.75 ± 3.62	38.43 ± 10.41	25.41 ± 13.96	38.26 ± 11.80	78.50 ± 11.37	95.96 ± 4.30	100.00 ± 0.00

**Table 7:** Results on COVID-SC testing with four levels of severity: P_NORMAL, P_MILD, P_MODERATE and P_SEVERE.

	Specificity	N_Precision	N_F1	Sensitivity	P_Precision	P_F1	Accuracy
ResNet-50	73.73 ± 5.07	79.46 ± 1.72	76.45 ± 3.40	71.62 ± 2.07	64.81 ± 4.89	67.94 ± 3.24	72.88 ± 3.40
ours	**77.95** ± **5.14**	**79.93** ± **4.78**	**78.64** ± **1.68**	**91.42** ± **3.07**	**90.80** ± **1.73**	**91.05** ± **0.92**	**87.40** ± **1.10**

Bold values indicates the best results for each metric.

We also compare our model with state-of-the-art automatic triage systems for COVID-19 based on CXR images on the COVIDGR-1.0 dataset^19^. Among the specific architectures designed for COVID-19 diagnosis we also include a generic ResNet-50 as a baseline. This network is fed with the whole image—*ResNet-50 without seg.* in Table 8—, and with the region of the lungs calculated using a lung segmentation network—*ResNet-50 with seg.* in Table 8. Moreover, we provide the results of two different versions of our approach to assess the impact of the support size in the model performance. Specifically, a 5- and 10-shot setups (5 and 10 images per category, respectively) are tested.

Table 8 shows how our approach generally achieves the best results on COVIDGR-1.0 with an accuracy of 77.35% on average using a support set with 5 images per category and 79.10% if the support set is increased up to 10 images per category. It improves the previous best model, COVID-SDNet, by 1.17% and 3.00% respectively. In terms of sensitivity, which is a key metric for every triage method, our method with the 10-shot setup outperforms every other previous method by a large margin, ranging the differences from 11.37% (COVID-SDNet) to 37.14% (COVIDNet-CXR).

**Table 8:** Results on COVIDGR-1.0 testing with four levels of severity: P_NORMAL, P_MILD, P_MODERATE and P_SEVERE.

	Specificity	N_Precision	N_F1	Sensitivity	P_Precision	P_F1	Accuracy
COVIDNet-CXR^26^	**88.82 ± 0.90**	3.36 ± 6.15	73.31 ± 3.79	46.82 ± 17.59	81.65 ± 6.02	56.94 ± 15.05	67.82 ± 6.11
COVID-CAPS^31^	65.74 ± 9.93	65.62 ± 3.98	65.15 ± 5.02	64.93 ± 9.71	66.07 ± 4.49	64.87 ± 4.92	65.34 ± 3.26
ResNet-50 without seg.^19^	79.87 ± 8.91	71.91 ± 3.12	75.40 ± 4.91	68.63 ± 6.08	78.75 ± 6.31	72.69 ± 3.45	74.25 ± 3.61
ResNet-50 with seg.^19^	78.41 ± 7.09	73.36 ± 4.66	75.46 ± 2.97	70.80 ± 8.26	77.17 ± 4.79	73.40 ± 4.01	74.60 ± 2.93
FuCiTNet^50^	80.79 ± 6.98	72.00 ± 4.48	75.84 ± 3.19	67.90 ± 8.58	78.48 ± 4.99	72.35 ± 4.76	74.35 ± 3.34
COVID-SDNet^19^	79.76 ± 6.19	74.74 ± 3.89	76.94 ± 2.82	72.59 ± 6.77	78.67 ± 4.70	75.71 ± 3.35	76.18 ± 2.70
ours (5-shot)	83.05 ± 7.60	75.22 ± 4.93	78.51 ± 2.46	71.64 ± 8.84	**81.67** ± **5.70**	75.73 ± 3.71	77.35 ± 2.45
ours (10-shot)	75.12 ± 6.50	**85.30** ± **3.72**	**79.68** ± **3.88**	**83.96** ± **4.87**	73.80 ± 4.87	**78.37** ± **3.14**	**79.10** ± **3.41**

Bold values indicates the best results for each metric.

Table 9 shows the comparison of the best previous method, COVID-SDNet, and the same two versions of our approach presented in Table 8. The results are presented independently for each severity level in the COVIDGR-1.0 dataset. Both 5- and 10-shot configurations of our network outperform COVID-SDNet in every severity level. Both methods achieve better results in patients with moderate and severe conditions, decreasing the accuracy in patients with mild severity. Nevertheless, our method outperforms COVID-SDNet in this challenging case by more than 10% with the 5-shot setup and by more than 16% with the 10-shot setup.

**Table 9:** Results by severity level on COVIDGR-1.0.

	COVID-SDNet^19^	Ours (5-shot)	Ours (10-shot)
P_NORMAL	–	31.82 ± 18.25	41.63 ± 13.13
P_MILD	46.00 ± 7.10	56.20 ± 16.20	62.40 ± 9.91
P_MODERATE	85.38 ± 1.85	86.08 ± 7.15	89.38 ± 6.18
P_SEVERE	97.22 ± 1.86	98.47 ± 3.25	99.50 ± 1.70

## Discussion and conclusions

COVID-19 has had a big impact both in population health and the global economy. Since the pandemic outbreak, the governments have put a lot of effort into stopping the spread of the virus, which has been carried out by mobility restrictions, health security protocols, increasing the detection rate of asymptomatic patients and improving the workflow of health services. In this regard, triage plays a crucial role in the optimization of health resources. To avoid the overcrowding of health centers, some options were made available to the population, such as hotlines or drive-through testing. However, when the symptoms are more pronounced and the patient potentially requires medical care, in-hospital triage is needed.

Given that the affection for COVID-19 in symptomatic patients is radiologically observable in the lungs, imaging techniques are widely used as a reliable triage system^51^. Among the several X-ray systems that are currently developed, CXR images are the main election regarding triage, mainly because of the low cost and the portability of the acquisition devices. Nevertheless, the evaluation of an X-ray image is a time-consuming process, as an experienced radiologist has to perform a thorough examination and identify features that are in line with the imaging spectrum of COVID-19. The automation of this process can, thus, lead to a better use of human resources and so a better triage procedure.

In this work, we developed a fully automatic approach to detect COVID-19 in CXR images. It is based on few-shot learning and so enables for high performance with small datasets, which is the case of every newly discovered condition such as COVID-19^17^. On top of a base few-shot topology, we proposed three main additions with a view to adapt the learning workflow to this specific domain and thus improve the performance. Specifically, we included a region proposal network to force the detector to focus only on lung areas. This was achieved by combining a lung segmentation network and a random patch generator. Furthermore, we proposed a combination of support sets to yield multiple estimations per image and then combine them through a weighted voting mechanism. Finally, we leveraged the severity scores provided in the assessed datasets to treat the COVID-19 detection as a multiclass problem, and included specific classification penalties to model the clinical misdiagnosis costs.

To assess the performance of this approach, we used the COVIDGR public database^19^, which contains more than 800 images. In addition, we developed COVID-SC, a database of 1,092 CXR images, comprising 439 negative and 653 positive images according to the RT-PCR results. To the best of our knowledge, this database entails the most realistic clinical scenario for several reasons: it contains images acquired with the same type of portable device; it includes not only the RT-PCR diagnosis, but also the severity according to the RALE score, which allows for a fine-grained analysis; as opposed to databases that only contain normal CXR in the negative class^19,21,23,24^, it provides images belonging to patients affected by other observable diseases, classified into those related to COVID-19 and those which are not.

Every contribution was analyzed individually to assess the performance impact in the COVIDGR public database. The lung-aware region proposals and the misdiagnosis-sensitive learning helped to improve the results by a significant margin, both in terms of overall accuracy and standard deviation. Although the detection of the lung region has already been used by other authors as a preprocessing step^19,36^, we incorporated a more elaborated lung patch generation module logic into the few-shot architecture so it can work directly with the raw images.

The addition of multiple support sets also yielded a significant improvement with respect to the baseline model. This represents a new paradigm of ensembling approaches. Instead of combining different heterogeneous models—which has already led to successful COVID-19 detection methods^34,35^—, our method can be seen as a data ensembling procedure, which consists in the execution of multiple comparisons against different class prototypes by using the same model. This produced not only an overall accuracy improvement, but also a dramatic reduction of the diagnosis variability.

The proposed method was compared with other approaches in the same public dataset^19^. Our method produced the most balanced performance and the best overall accuracy. Furthermore, the reported performance across the different COVID-19 severity levels confirms that the proposed method outperforms the best previous model in every case. It is also worth noting that the greatest difference is achieved in the mild cases, whose detection is crucial in a triage process^52^.

We leveraged the wide range of categories present in the COVID-SC database to assess the performance of the proposed method in the most realistic scenario. Under the most favorable setting—negative images with no observable condition, and mild, moderate and severe positive images—, the overall accuracy was 87.4%. As expected, COVID-related and non-related diseases hindered the detection of negatives. However, every triaging system is required to detect as many positive patients as possible, so it is preferable to lose negative detections if positive patients are better detected. In the same way, images belonging to positive yet asymptomatic patients worsened the detection of positives, which is clinically consistent given that even expert radiologists are not able to diagnose a positive patient if there are no radiologic findings.

The reported results also pointed out the noticeable performance difference of the same method in two different datasets, even if the databases are well documented, large, and presumably not biased regarding the acquisition device or the severity of the patients. In this regard, future work should involve the validation of the proposed approach in a wider corpus of CXR databases through a cross-dataset validation scenario, that is, the assessment of the performance obtained in datasets different from the ones used for the training process.

Moreover, the use of lung patches to make the predictions could be exploited to develop a visual explanation mechanism, in which heatmaps can be generated to highlight those regions that contribute the most to the classification outcome. This would help the experts to easily check the behavior of the automatic classifier and validate it more thoroughly by comparing the heatmaps to clinical findings reported in the literature.

In conclusion, we proposed an accurate and fully automatic method for detecting COVID-19, which is based on deep learning and few-shot techniques. It was validated in a public database, outperforming the previous proposed methods in terms of detection accuracy. Furthermore, we developed COVID-SC, a novel CXR database with more than 1000 images organized into three non-COVID categories and four COVID categories, with a view to representing a realistic triage scenario. We also provided baseline results for the proposed system in this database, concluding that it could be useful for triage procedures and patient follow-up.

## Data Availability

The COVID-SC database is publicly available under request at https://citius.usc.es/t/covid-sc.

## References

[1] Chen J (2020). Pathogenicity and transmissibility of 2019-nCoV-a quick overview and comparison with other emerging viruses. Microb. Infect..

[2] Morens, D. M., Folkers, G. K. & Fauci, A. S. The concept of classical herd immunity may not apply to COVID-19. *J. Infect. Dis.* (2022). 10.1093/infdis/jiac109 PMC9129114 35356987

[3] Rostami A (2020). SARS-CoV-2 seroprevalence worldwide: A systematic review and meta-analysis. Clin. Microbiol. Infect..

[4] Wilson ME, Chen LH (2020). Travellers give wings to novel coronavirus (2019-nCoV). J. Travel Med..

[5] Shen M (2020). Recent advances and perspectives of nucleic acid detection for coronavirus. J. Pharm. Anal..

[6] Cascella, M., Rajnik, M., Aleem, A., Dulebohn, S. C. & Di Napoli, R. Features, evaluation, and treatment of coronavirus (COVID-19). *Statpearls [internet]* (2022). 32150360

[7] Jin KN (2022). Korean clinical imaging guidelines for justification of diagnostic imaging study for COVID-19. J. Korean Soc. Radiol..

[8] Huang C (2020). Clinical features of patients infected with 2019 novel coronavirus in Wuhan, China. Lancet.

[9] Guan W-J (2020). Clinical characteristics of coronavirus disease 2019 in China. New Engl. J. Med..

[10] Kim, H., Hong, H. & Yoon, S. H. Diagnostic performance of CT and reverse transcriptase-polymerase chain reaction for coronavirus disease 2019: A meta-analysis. *Radiology* 201343 (2020). 10.1148/radiol.2020201343 PMC7233409 32301646

[11] Wong HYF (2020). Frequency and distribution of chest radiographic findings in COVID-19 positive patients. Radiology.

[12] Schiaffino S (2020). Diagnostic performance of chest X-ray for COVID-19 pneumonia during the SARS-CoV-2 pandemic in Lombardy, Italy. J. Thorac. Imag..

[13] Shi F (2020). Review of artificial intelligence techniques in imaging data acquisition, segmentation and diagnosis for COVID-19. IEEE Rev. Biomed. Eng..

[14] Fernández-Miranda PM (2021). Developing a training web application for improving the COVID-19 diagnostic accuracy on chest X-ray. J. Digit. Imaging.

[15] Cohen, J. P. *et al.* COVID-19 image data collection: Prospective predictions are the future. arXiv:2006.11988 (2020).

[16] Wang L, Lin ZQ, Wong A (2020). Covid-net: A tailored deep convolutional neural network design for detection of COVID-19 cases from chest X-ray images. Sci. Rep..

[17] Roberts M (2021). Common pitfalls and recommendations for using machine learning to detect and prognosticate for COVID-19 using chest radiographs and CT scans. Nat. Mach. Intell..

[18] Jadon S (2021). COVID-19 detection from scarce chest X-ray image data using few-shot deep learning approach. Proc. SPIE.

[19] Tabik S (2020). COVIDGR dataset and COVID-SDNet methodology for predicting COVID-19 based on chest X-ray images. IEEE J. Biomed. Health.

[20] Chowdhury ME (2020). Can AI help in screening viral and COVID-19 pneumonia?. IEEE Access.

[21] Signoroni A (2021). BS-Net: Learning COVID-19 pneumonia severity on a large chest X-ray dataset. Med. Image Anal..

[22] Tsai, E. *et al.* Data from medical imaging data resource center (MIDRC)—RSNA international COVID radiology database (RICORD) release 1c-chest X-ray, Covid+ (MIDRC-RICORD-1c). 10.7937/91ah-v663 (2021).

[23] Winther, H. B. *et al.* COVID-19 image repository. 10.6084/m9.figshare.12275009.v1 (2020).

[24] Wang, L. *et al.* Actualmed COVID-19 chest X-ray dataset initiative. https://github.com/agchung/Actualmed-COVID-chestxray-dataset (2020).

[25] Wang, L. *et al.* Figure 1 COVID-19 chest X-ray dataset initiative. https://github.com/agchung/Figure1-COVID-chestxray-dataset (2020).

[26] Wang L, Lin ZQ, Wong A (2020). COVID-Net: A tailored deep convolutional neural network design for detection of COVID-19 cases from chest X-ray images. Sci. Rep..

[27] de la Iglesia Vayá, M. *et al.* BIMCV COVID-19+: A large annotated dataset of RX and CT images from COVID-19 patients. arXiv:2006.01174 (2020).

[28] Wong HYF (2020). Frequency and distribution of chest radiographic findings in patients positive for COVID-19. Radiology.

[29] Nishio M, Noguchi S, Matsuo H, Murakami T (2020). Automatic classification between COVID-19 pneumonia, non-COVID-19 pneumonia, and the healthy on chest X-ray image: combination of data augmentation methods. Sci. Rep..

[30] Khan AI, Shah JL, Bhat MM (2020). CoroNet: A deep neural network for detection and diagnosis of COVID-19 from chest X-ray images. Comput. Methods Prog. Biol..

[31] Afshar P (2020). COVID-CAPS: A capsule network-based framework for identification of COVID-19 cases from X-ray images. Pattern Recogn. Lett..

[32] Ouchicha C, Ammor O, Meknassi M (2020). CVDNet: A novel deep learning architecture for detection of coronavirus (COVID-19) from chest X-ray images. Chaos Solitons Fractals.

[33] Rahman T (2021). Exploring the effect of image enhancement techniques on COVID-19 detection using chest X-ray images. Comput. Biol. Med..

[34] Gupta A, Gupta S, Katarya R (2021). InstaCovNet-19: A deep learning classification model for the detection of COVID-19 patients using chest X-ray. Appl. Soft. Comput..

[35] Wang, N., Liu, H. & Xu, C. Deep learning for the detection of COVID-19 using transfer learning and model integration. In *IEEE International Conference on Electronics Information and Emergency Communication*, 281–284 (IEEE, 2020).

[36] Rajaraman S (2020). Iteratively pruned deep learning ensembles for COVID-19 detection in chest X-rays. IEEE Access.

[37] Oh Y, Park S, Ye JC (2020). Deep learning COVID-19 features on CXR using limited training data sets. IEEE Trans. Med. Imaging.

[38] Kang H (2020). Diagnosis of coronavirus disease 2019 (COVID-19) with structured latent multi-view representation learning. IEEE Trans. Med. Imaging.

[39] Waheed A (2020). CovidGAN: Data augmentation using auxiliary classifier GAN for improved COVID-19 detection. IEEE Access.

[40] Loey M, Smarandache F, M Khalifa NE (2020). Within the lack of chest COVID-19 X-ray dataset: A novel detection model based on GAN and deep transfer learning. Symmetry.

[41] Vinyals O, Blundell C, Lillicrap T, Wierstra D (2016). Matching networks for one shot learning. Adv. Neural Inf..

[42] Cui H, Wei D, Ma K, Gu S, Zheng Y (2020). A unified framework for generalized low-shot medical image segmentation with scarce data. IEEE Trans. Med. Imaging.

[43] Shorfuzzaman M, Hossain MS (2021). MetaCOVID: A siamese neural network framework with contrastive loss for n-shot diagnosis of COVID-19 patients. Pattern Recogn..

[44] Teixeira LO (2021). Impact of lung segmentation on the diagnosis and explanation of COVID-19 in chest X-ray images. Sensors.

[45] Tartaglione E, Barbano CA, Berzovini C, Calandri M, Grangetto M (2020). Unveiling COVID-19 from chest X-ray with deep learning: A hurdles race with small data. Int. J. Environ. Res. Public Health.

[46] Rubner Y, Tomasi C, Guibas LJ (2000). The earth mover’s distance as a metric for image retrieval. Int. J. Comput. Vis..

[47] Ronneberger, O., Fischer, P. & Brox, T. U-net: Convolutional networks for biomedical image segmentation. In *Proceedings of MICCAI* 234–41 (2015).

[48] Jaeger S (2014). Two public chest X-ray datasets for computer-aided screening of pulmonary diseases. Quant. Imag. Med. Surg..

[49] Shiraishi J (2000). Development of a digital image database for chest radiographs with and without a lung nodule: Receiver operating characteristic analysis of radiologists’ detection of pulmonary nodules. Am. J. Roentgenol..

[50] Rey-Area M, Guirado E, Tabik S, Ruiz-Hidalgo J (2020). FuCiTNet: Improving the generalization of deep learning networks by the fusion of learned class-inherent transformations. Inform. Fusion.

[51] Kaufman AE (2020). Review of radiographic findings in COVID-19. World J. Radiol..

[52] Peros G (2020). Organizing a COVID-19 triage unit: A Swiss perspective. Emerg. Microbes Infect..

